# Supplementary material for: Winners and losers from Pfizer and Biontech’s vaccine announcement: Evidence from S&P 500 (Sub)sector indices
Source: PLoS One. 2022 Oct 14;17(10):e0275773. doi: 10.1371/journal.pone.0275773 (PMC9565377; doi:10.1371/journal.pone.0275773)
Supplement: Attachment — Submitted filename: Referee_report_PONE-D-22-14074_.docx [file pone.0275773.s002.docx]

Dear Author/s

I would like to thank the author(s) for your submission and appreciate the opportunity to read and review your manuscript. I enjoyed reading it. My comments concern only with clarity, text, methodology used and interpretation of results. I would recommend publication, after the issues below are addressed.

**Changes which must be made before publication:**

**Minor changes:**

1. **Abstract**: *please expand the abstract and add the methodology used.*
2. **The Keywords:** the keywords does not accurately reflect the content of the study, additional 2-3 keywords should be added.
3. **The introduction section**: I think it is good.
4. **The literature review section**: *please expand the literature review and add some latest references.*
5. **The methodology section**: It's okay, but it could be better explained and motivated further. Please reference all numbered equations in text. Currently, numbered equations [1, 2, 3, 4] in the manuscript have not been cited in text.
6. **The empirical results and discussion section:** the results and discussion section should be better analyzed and developed further.
7. **Conclusion section:** It's okay, but it could be better explained and motivated with a few more minor adjustments, please try to augment its quality with more in-depth investigation and analysis:
   a. I recommend to change the section title to (Conclusion and Policy Recommendations).
   b. "limitations, and further research" could be added
8. **The language of the paper:** the language of the paper needs a careful editing by a native speaker.

**Additional comments:** the author/s are recommended to do the following to increase the paper readability.

1. To start with, the paper should more clearly and more explicitly spell out its objectives.
2. Develop the literature review section of the article to include 3-5 latest journal references (2021-2022) and relevant extracts from them.
3. Please, avoid placing tables or figures before their first mention in the text, and the analysis should always be below the figure or the table.
4. Please avoid the following word/s; "our analysis" (please see p. 5), "our results" (please see p.8) , "our earlier results" (please see p.9). Please replace with "the present study………" or "the current study………"…etc.
5. Please be careful in using the abbreviations and abbreviated words throughout the paper.
6. Please be careful in using "Punctuating", e.g. (comma, full stop, etc…) throughout the paper.
