# Supplementary material for: Winners and losers from Pfizer and Biontech’s vaccine announcement: Evidence from S&P 500 (Sub)sector indices
Source: PLoS One. 2022 Oct 14;17(10):e0275773. doi: 10.1371/journal.pone.0275773 (PMC9565377; doi:10.1371/journal.pone.0275773)
Supplement: Attachment — Submitted filename: Pfizer paper review report.docx [file pone.0275773.s003.docx]

**Winners and Losers from Pfizer and Biontech's Vaccine Announcement: Evidence from S&P 500 (Sub)Sector Indices**

**Reviewer Comments**

In the underlined research topic “*Winners and Losers from Pfizer and Biontech's Vaccine Announcement: Evidence from S&P 500 (Sub)Sector Indices”* is a novel part to the existing literature. The title of the article is remarkable, the presentation is good and the article may provide several scientific justifications. Below are several suggestions which will increase writer input to technical research and probability.

1. The paper needs to be improved in all parts (justification of the contribution, conceptual background, the method). I hope you find the reviewers' comments helpful in developing the paper further.

2. A single paragraph serves as the article's assessment of the literature, and the commentary is disorganized and illogical. I'd advise the writers to restructure the literature review section and make the logical connections clearer.

3. Globally, the manuscript needs a revision of the English and there are paragraphs and sentences written in a very confusing way.

4. The technique used in the study is appropriate for the findings, and but the authors should review it again to make is more attractive.

5. At the bottom of the table, provide a note identifying the data source.

6. The author should expand on the empirical findings.

7. To make the paper statistically strong, the authors should need to add more innovative techniques to make the results much clear.

8. The results follow the methodology but the interpretation of tables and graphs need more quality presentation.

9. The results and discussion of the study is quite lengthy despite the importance of the information but if possible to eliminate some of the secondary information that does not disturb the quality of the research flow.

10. A separate set of data is needed for the author.

11. To write an equation, the author must adhere to a prescribed format.

12. "Illustration from simulation" is the desired outcome of the simulation research.

13. The author must distinguish the inference from the conclusion in order to avoid confusion.

15. Referencing should follow a standard structure that is consistent with the references mentioned in journal authors’ guidelines.

16. Discussions should add some latest references of 2021 and 2022 to prove the results.

17. More explicitly stated restrictions and future proposals should be included in this article.

Finally, it’s a nice attempt, the topic is of interest and has a wide range of policy implementations. Bases on the above-mentioned comments, I suggest “**major revision**” of the article for more practicability before **“publication”**.
