# Supplementary material for: Winners and losers from Pfizer and Biontech’s vaccine announcement: Evidence from S&P 500 (Sub)sector indices
Source: PLoS One. 2022 Oct 14;17(10):e0275773. doi: 10.1371/journal.pone.0275773 (PMC9565377; doi:10.1371/journal.pone.0275773)
Supplement: Attachment — Submitted filename: Referee_report_PONE-D-22-14074-R2.docx [file pone.0275773.s005.docx]

Dear Author(s)

I would like to thank the author(s) for addressing my initial comments. The author(s) have greatly improved their manuscript and responded to the points that I have raised. Following the revision to the paper, some of my additional "minor comments" relate to some of the amendments made. My remaining comments concern only with clarity, text, and methodology. I would recommend publication, after the issues below are addressed.

**Minor changes:**

1. **The introduction section**: Some of the terminology used in the manuscript needs to be unified. For instance, the use of two different words that have the same meaning interchangeably could confuse some readers. Dear author(s)please see the introduction section, p. 3 line 16-17 the author(s) mentioned that " "*Section 5 presents empirical findings and discussion, and Section 6 concludes*", while in p. 9 line 9 the author(s)used another words "*5 Empirical results and Discussion*", then in p. 14 line 1 the author(s)used another words "6 Conclusion and Policy Recommendations".
2. **The Literature review section**: In the first paragraph the author(s) mentioned that "*With the outbreak of the coronavirus (COVID-19) pandemic, an increasing number of researchers have examined the impact of the pandemic on international financial markets. Towards this end, recent studies have established that the pandemic impacted stock markets (Acharya et al., 2021; Al-Awadhi et al., 2020; Baek et al., 2020; Engelhardt et al., 2021; Kapar et al., 2021; Kucher et al., 20221; Rouatbi et al., 2021), bond markets (Augustin et al., 2022; Chen et al., 2021; Falato et al., 2021; Haddad et al., 2021), gold markets (Corbert et al., 2020; Gharib et al. 2021; Mensi et al., 2020), exchange rate (Li et al., 2021; Aquilante et al., 2022; Jamal and Bhat, 2022), crude oil markets (Sharif et al., 2020; Mensi et al., 2020; Shaikh, 2021; Gharib et al., 2021; Wang et al., 2022), cryptocurrency markets (Corbert et al., 2020; Conlon and McGee, 2020; Yarovaya et al., 2020; Vidal-Tomás, 2021; Hong and Yoon, 2022), real estate markets (Balemi et al., 2021; Ling et al., 2020; Milcheva et al., 2022), and other asset classes*". It contains too a large number of references, I would suggest you devote some effort to discuss such previous studies and – more importantly – their relationships and the major factors responsible for differences in results from same study conducted by different researchers.
3. **The methodology section**: The description of the methods is much clearer now. But I would suggest the author(s)to add a new subtitle after the methodology section title. Please add a subtitle (**4.1 Event Study Methodology**) then add a single paragraph discussing the theoretical basis of this methodology to help the reader to follow the section better.

Finally, based on the above-mentioned comments, I suggest “minor revision” of the paper before publication.
