# Supplementary material for: Winners and losers from Pfizer and Biontech’s vaccine announcement: Evidence from S&P 500 (Sub)sector indices
Source: PLoS One. 2022 Oct 14;17(10):e0275773. doi: 10.1371/journal.pone.0275773 (PMC9565377; doi:10.1371/journal.pone.0275773)
Supplement: Attachment — Submitted filename: Response to Reviewers-Second One.docx [file pone.0275773.s006.docx]

September 7, 2022

First of all, we would like to express our gratitude for your careful reading and comments on

our manuscript. They have helped to clarify important aspects of the manuscript that were unclear in the previous version. We worked on these comments and believe our work benefited as a result. In this document, we provide our response to each of the points raised in your referee report.

**Minor changes:**

1. **The introduction section**: Some of the terminology used in the manuscript needs to be unified. For instance, the use of two different words that have the same meaning interchangeably could confuse some readers. Dear author(s)please see the introduction section, p. 3 line 16-17 the author(s) mentioned that " "*Section 5 presents empirical findings and discussion, and Section 6 concludes*", while in p. 9 line 9 the author(s)used another words "*5 Empirical results and Discussion*", then in p. 14 line 1 the author(s)used another words "6 Conclusion and Policy Recommendations".

**Answer:** This is corrected. In Page 3, we mentioned as this: *Section 2 discusses the literature review; Section 3 presents the data; Section 4 explains the methodology; Section 5 presents empirical results and discussion; and Section 6 concludes and presents policy recommendations.*

2. **The Literature review section**: In the first paragraph the author(s) mentioned that "*With the outbreak of the coronavirus (COVID-19) pandemic, an increasing number of researchers have examined the impact of the pandemic on international financial markets. Towards this end, recent studies have established that the pandemic impacted stock markets (Acharya et al., 2021; Al-Awadhi et al., 2020; Baek et al., 2020; Engelhardt et al., 2021; Kapar et al., 2021; Kucher et al., 20221; Rouatbi et al., 2021), bond markets (Augustin et al., 2022; Chen et al., 2021; Falato et al., 2021; Haddad et al., 2021), gold markets (Corbert et al., 2020; Gharib et al. 2021; Mensi et al., 2020), exchange rate (Li et al., 2021; Aquilante et al., 2022; Jamal and Bhat, 2022), crude oil markets (Sharif et al., 2020; Mensi et al., 2020; Shaikh, 2021; Gharib et al., 2021; Wang et al., 2022), cryptocurrency markets (Corbert et al., 2020; Conlon and McGee, 2020; Yarovaya et al., 2020; Vidal-Tomás, 2021; Hong and Yoon, 2022), real estate markets (Balemi et al., 2021; Ling et al., 2020; Milcheva et al., 2022), and other asset classes*". It contains too a large number of references, I would suggest you devote some effort to discuss such previous studies and – more importantly – their relationships and the major factors responsible for differences in results from same study conducted by different researchers.

**Answer:** We have updated this paragraph and discuss the papers that talks about the effect of COVID-19 only on stock markets in details as our paper is related to the stock market. The additional two paragraphs are as below:

With the outbreak of the coronavirus (COVID-19) pandemic, an increasing number of researchers have examined the impact of the pandemic on stock markets. Baker et al. (2020) document that no previous infectious disease outbreak, including the Spanish Flu, has impacted the stock market as forcefully as the COVID-19 pandemic due to strict government restrictions on commercial activity and voluntary social distancing. Exploring the direct effects and spillovers of COVID-19, He et al. (2020a) find that COVID-19 has a negative but short-term impact on stock markets of affected countries. By using a large sample of 63 stock markets covering all key markets, Kapar et al. (2021) find that the Wuhan lockdown induces negative spillover effects on markets in Europe, North America and other global markets. This is mainly attributed to fear and uncertainty as these markets had yet to introduce domestic restrictions and had minimal infections at the time. The rapid transmission of cases outside China particularly in Europe and the introduction of containment measures result in severe market decline which highlights the need for quick, globally coordinated response to contagious diseases.

Controlling for traditional market drivers (such as investor sentiment, credit risk, liquidity risk, safe-haven asset demand and the price of oil), O’Donnell et al. (2021) conclude that the daily total count of confirmed COVID-19 cases is a leading factor in influencing equity prices. Using panel data analysis, Al-Awadhi et al. (2020) estimate that both the daily growth in confirmed cases and number of deaths caused by COVID-19 have significant negative effects on returns in Chinese stock market. Ashraf (2020) finds that stock markets react more strongly to the growth in number of confirmed cases as compared to the growth in number of deaths. Mazur et al. (2021) examine the US stock market during the crash of March 2020. They estimate that approximately 90% of the S&P 1500 stocks generate asymmetrically distributed large negative returns. The consensus from this emerging literature on COVID-19 suggests that stock markets respond negatively and significantly to COVID-19. Individual stock responses may vary, however, depending on several factors.

3. **The methodology section**: The description of the methods is much clearer now. But I would suggest the author(s)to add a new subtitle after the methodology section title. Please add a subtitle (**4.1 Event Study Methodology**) then add a single paragraph discussing the theoretical basis of this methodology to help the reader to follow the section better.

**Answer:** 4.1 Event Study Methodology subtitle is included under Methodology section. A paragraph is added to discuss the theoretical basis of this methodology.
